# Prevalence of HIV, HBV, and HCV and Related Risk Factors amongst Male Homeless People in Lorestan Province, the West of Iran

**Published:** 2017-03-11

**Authors:** Amin Doosti-Irani, Hamid Mokhaeri, Ali Chegini Sharafi, Mohammad Reza Aghasadeghi, Morteza Hajimiragha, Mohammad Saki, Mohammad Hassan Kayedi, Ehsan Mostafavi

**Affiliations:** ^1^ Department of Epidemiology, School of Public Health, Hamadan University of Medical Sciences, Hamadan, Iran; ^2^ Deputy of Health, Lorestan University of Medical Sciences, Khorramabad, Iran; ^3^ Department of Hepatitis and AIDS, Pasteur Institute of Iran, Tehran, Iran; ^4^ Department of Parasitology, Faculty of Medicine, Lorestan University of Medical Sciences, Khorramabad, Iran; ^5^ Department of Epidemiology and Biostatistics, Research Centre for Emerging and Reemerging Infectious Diseases, Pasteur Institute of Iran, Tehran, Iran

**Keywords:** Homeless People, HIV, Hepatitis B, Hepatitis C, Prevalence, Iran

## Abstract

**Background:** Homeless people are at a higher risk of blood-born infectious diseases. The aim of this study was to estimate the prevalence of HIV, HBV, HCV and related risk factors among male homeless people.

**Study design:** A cross-sectional study.

**Methods:** This study was conducted in Khorramabad City, western Iran from January to June 2015. A pre-designed validated questionnaire was used to collect the data on behavioral and other potential risk factors. Blood samples were taken in order to diagnose HIV, hepatitis B and C infections. The prevalence of HIV, hepatitis B, C and related risk factors was reported with a 95% confidence interval (CI).

**Results:** The participants were 307 male homeless people with a mean (±SD) age of 35.86 (±9.62) yr. The prevalence of HIV, HBs Ag, and HCV Ab positive cases was 6.51% (95% CI: 4.23, 9.90), 0.98% (95% CI: 0.31, 3.00), and 31.27% (26.31, 36.71), respectively. The prevalence of co-infections of HIV and HCV Ab+ was 5.76% (95% CI: 1.34, 8.51). The most common recently used drugs were heroin, methamphetamine, and opium with a prevalence of 44.30%, 41.04%, and 24.76%, correspondingly.

**Conclusions:** According to this study, prevalence of HIV and hepatitis C among homeless people was considerable. Abusing heroin, methamphetamine, and industrial drugs was also significant. Considering the association between drug abuse, HIV, and hepatitis C infections, planning for effective control and preventive interventions is important in homeless people.

## Introduction

Homeless people are those without any house or permanent shelter for sleeping. They live in the corners of streets, parks or public places and locations assigned by governmental or non-governmental organizations^1^. Homelessness leads to an increase in social and public health problems in the world. Homeless phenomenon is associated with behavioral, social, and environmental risk factors ^2^. Furthermore, homeless people are at a higher risk of blood-born infectious diseases, such as HIV/AIDS, hepatitis B, and C, because of risky sexual behaviors, injecting drug usage, financial and nutritional poverty, and low literacy level^3, 4^. Additionally, homeless people’s access to health and medical care is low because of extreme poverty, so the rate and severity of infectious diseases among them are higher than the general population. Homeless may spread disease amongst them and therefore endanger the population ^5^. In addition, the mortality rate among these people is approximately four times more than the general population is ^6, 7^.

Globally, the prevalence of HIV, tuberculosis, and hepatitis C was reported as 0.3-21.1, 0.2-7.7, and 3.9-36.2%, in homeless people, respectively ^8^. The prevalence of HIV, hepatitis B virus (HBV), and hepatitis C virus (HCV) among homeless people was reported as 3.4%, 2.6%, and 23.3%, respectively in Tehran, the capital of Iran ^9^. This number was also reported as 19%, 6%, and 56% among the male injection-drug users in Arak the capital of Markazi Province of Iran, in 2012, in that order^10^.

Due to the lack of information on the prevalence of blood-born infectious diseases among homeless people in the west of Iran, this study was conducted to estimate the prevalence of HIV, HBV, and HCV and the related risk factors among male homeless people in Lorestan Province, Western Iran.

## Methods

This cross-sectional study was conducted in Khorramabad City, the capital of Lorestan Province in the west of Iran. The population of the city, according to the national census in 2011, was about five hundred thousand ^11^.

The proposal of this study was approved by the Board of Ethics Committee, Pasteur Institute of Iran (number: 94.0201.6023). All the participants enrolled voluntarily into the study and their informed consent was obtained.

The participants of this study were recruited from Khorramabad City from January to June 2015. All male homeless people in the city (345 people) were invited to participate the study, but eventually, 307 homeless (response rate=89%) participated.

The inclusion criteria for participants were 1) being continuously or discontinuously homeless in the month before the study for at least ten days, and 2) if they had informed consent to participate in the study. After receiving the informed consent, the trained interviewers visited and interviewed the homeless people in a quiet location and during a face-to-face interview; the questionnaire was completed. The participants received the free health care service with methadone therapy in an addiction treatment center in Khorramabad.

A pre-designed validated questionnaire^9^ was used to collect the data on behavioral and other potential risk factors. The questionnaire included socio-demographic characteristics, potential risk factors for blood-borne infections such as a history of high-risk sexual activity, substance abuse, and a history of criminal behaviors or jail. Ten ml blood samples were taken in order to diagnose HIV, HCV, and HBV infections.

Diagnosis of HIV was performed using ELISA test. The positive samples were tested again using ELISA. People with two positive ELISA tests were referred to a counseling center for final confirmation of HIV and receiving treatment. Blood samples were tested in order to determine hepatitis B (HBsAg) and HCV antibody using commercially available kits (DIA PRO Diagnostic Bioprobes, Srl., Italy). People with positive tests for hepatitis B or hepatitis C were referred for receive treatment.

The prevalence of HIV, HBV, and HCV and the related risk factors was reported with a 95% confidence interval (CI). The association between the potential risk factors and infections was evaluated using logistic regression model. Hence, due to the small number of positive cases of HIV and HBV, the adjusted logistic regression model could not be used to assess the potential risk factors and adjust the confounding variables. However, the adjusted model was used to assess the association of potential risk factors with HCV and adjust the confounding variables. A *P*-value less than 0.20 in a univariate model was considered to include the variables in the adjusted model. The backward stepwise approach was used for final model building. Stata 11 (Stata Corp, College Station, TX, USA) was used for the data analysis.

## Results

In this cross-sectional study, 307 male homeless people with a mean (±SD) age of 35.9 (±9.6) yr were enrolled. The median age of participants was 34 yr. Three hundred and five (99.4%) participants had a history of drug abuse. The mean (±SD) age of the first drug use was 18.9 (±6.4) yr and the minimum and maximum ages of starting drug use were 7 and 58 yr, respectively. The majority of the participants (42.2%) had an intermediate level of education, while 6.2% were illiterate and 7.5% had an academic level of education. According to job status, 18.9% were workers and 16.69% were unemployed. A considerable proportion of the participants (44.3%) were single (Table 1).

**Table 1:** Demographic characteristics of the participants

**Variables**	**Number**	**Percent**
Education		
Illiterate	19	6.2
Primary	72	23.5
Intermediate	129	42.2
high school grad	63	20.6
Academic	23	7.5
Job		
Worker	58	18. 9
Unemployed	50	16.3
Welder	28	9.1
Driver	24	7.8
Self-employed	19	6.2
Salesman	18	5.9
Baker	13	4.2
Farmer	12	3.9
Construction worker	10	3.3
Other	75	24.4
Marriage status		
Married	113	36.8
Single	136	44.3
Divorced	52	16.9
Widow	6	2.0
Number of child		
None	166	54.1
One	68	22.2
Two	43	14.0
More than three	30	9.8
First drug used		
Opium	151	49.5
Hashish	65	21.3
Alcohol	50	16.4
Heroin	14	4.6
Alcohol & Hashish	9	3.0
Tramadol	2	0.7
Other	14	4.6

The prevalence of HIV, HBs Ag, and HCV Ab positive cases was 6.51% (95% CI: 4.23, 9.90), 0.98% (95% CI: 0.31, 3.00), and 31.27% (26.31, 36.71), respectively.

According to the history of drug used, opium with 94.1%, was the most frequently used drug and diphenoxylate with 21.2%, stood at the bottom of the list. Among the recently used drugs, heroin, methamphetamine, and opium with 44.3%, 41.0%, and 24.8% were the most commonly used drugs (Table 2). In addition, 38.76% of the participants had a history of injecting drug use and 70.4% had a history of illicit sex.

**Table 2:** The proportion of the history of drug used and recently used drug based on the type of drugs

**Type of drug**	**History of drug used**		**Recently used drug**	
	**Number**	**Percent**	**Number**	**Percent**
Hashish	208	67.8	5	1.6
Opium	289	94.1	76	24.8
Sap opium	225	73.3	7	2.3
Heroin	228	74.3	136	44.3
Temgesic	71	23.1	0	0.0
Methamphetamine	221	72.0	126	41.0
Diphenoxylate	65	21.2	0	0.0
Codeine	116	37.8	1	0.3
Methadone	231	75.2	9	2.9
Hypnotic	141	45.9	42	13.7
Tramadol	123	40.1	3	0.9

### HIV risk factors

According to the results of the unadjusted analysis (Table 3), the odds ratio (OR) for HIV positive cases amongst single homeless people compared with the married homeless people was 1.4 (0.5, 3.7). The use of temgesic (OR=4.6; 95% CI: 1.8, 11.7) and hypnotic (OR=12; 95% CI: 2.7, 52.7) was associated with HIV positive status. The history of sharing injection drugs was strongly associated with HIV (OR=92.3; 95% CI: 12.1, 705.7).

**Table 3:** Unadjusted analysis of potential risk factors of human immunodeficiency virus (HIV) and hepatitis C virus (HCV)

**Variables**	**HIV**			**HCV**		
	**Positive**	**Negative**	**OR (95% CI)**	**Positive**	**Negative**	**OR (95% CI)**
Marriage status						
Married	6	106	1.00	29	83	1.00
Single	14	181	1.37 (0.51, 3.67)	67	128	1.51 (0.89, 2.51)
Educational level						
Under diploma	16	204	1.00	71	149	1.00
Diploma and upper	4	82	0.62 (0.20, 1.92)	24	62	0.81 (0.47, 1.41)
Hashish						
No	4	95	1.00	15	84	1.00
Yes	16	192	1.98 (0.64, 6.08)	81	127	3.57 (1.93, 6.61)
Opium						
No	1	17	1.00	3	15	1.00
Yes	19	270	1.20 (0.19, 9.48)	93	196	2.37 (0.67, 8.40)
Sap opium						
No	5	77	1.00	16	66	1.00
Yes	15	210	1.10 (0.39, 3.13)	80	145	2.28 (1.24, 4.20)
Heroin						
No	0	79	1.00	7	72	1.00
Yes	20	208	No data	89	139	6.59 (2.90, 14.96)
Temgesic						
No	9	227	1.00	53	183	1.00
Yes	11	60	4.62 (1.83, 11.67)	43	28	5.30 (3.01, 9.34)
Methamphetamine						
No	3	83	1.00	11	75	1.00
Yes	17	204	2.31 (0.66, 8.08)	85	136	4.26 (2.14, 8.48)
Diphenoxylate						
No	14	228	1.00	67	175	1.00
Yes	6	59	1.66 (0.61, 4.50)	29	36	2.10 (1.20, 3.70)
Codeine						
No	9	182	1.00	41	150	1.00
Yes	11	105	2.12 (0.85, 5.28)	55	61	3.30 (2.00, 5.45)
Methadone						
No	1	75	1.00	13	63	1.00
Yes	19	212	6.72 (0.88, 51.08)	83	148	2.72 (1.41, 5.23)
Hypnotic						
No	2	164	1.00	23	143	1.00
Yes	18	123	12.00 (2.73, 52.69)	73	68	6.67 (3.84, 11.57)
Tramadol						
No	9	175	1.00	42	142	1.00
Yes	11	112	1.91 (0.77, 4.75)	54	69	2.64 (1.61, 4.34)
Injection drug use						
No	0	188	1.00	0	188	1.00
Yes	20	99	No data	96	23	No data
Sharing syringe						
No	1	238	1.00	32	207	1.00
Yes	19	49	92.29 (12.07, 705.67)	64	4	103.50 (35.26, 303.73)
History of Jail						
No	3	69	1.00	13	59	1.00
Yes	17	218	1.80 (0.51, 6.30)	83	152	2.49 (1.28, 4.78)
Illicit sex						
No	5	86	1.00	22	69	1.00
Yes	15	201	1.28 (0.45, 3.64)	74	142	1.63 (0.94, 2.85)
Condom use history						
No	7	146	1.00	42	111	1.00
Yes	12	126	1.98 (0.75, 5.20)	49	89	1.45 (0.88, 2.39)

### HCV risk factors

The unadjusted analysis showed that the use of hashish, sap opium, temgesic, methamphetamine, diphenoxylate, codeine, methadone, hypnotic, and tramadol increased the odds of HCV significantly. In addition, the history of sharing injection equipments and jail increased the odds of HCV by 103.5 (95% CI: 35.3, 303.7) and 2.5 (95% CI: 1.3, 4.3), respectively (Table 3).

In the adjusted model, the association between always condom use with HCV was protective OR=0.63 (95% CI: 0.23, 1.74), however it was not statistically significant. The use of codeine (OR=2.54; 95% CI: 1.28, 5.01), heroin (OR=3.61; 95% CI: 1.14, 11.41) and temgesic (OR=2.26; 1.11, 4.60) were significantly associated with HCV (Table 4).

**Table 4:** Multivariate analysis for potential risk factors of hepatitis C virus (HCV)

**Variables**	**HCV+**	**HCV-**	**OR (95% CI)**
Education			
Under diploma	71	149	1.00
Diploma and upper	24	62	0.78 (0.39, 1.53)
Illicit sex			
No	22	69	1.00
Yes	74	142	1.38 (0.57, 3.37)
Refer to Addiction Treatment Center	
No	12	73	1.00
Yes	84	138	2.02 (0.82, 4.95)
Condom use			
No	34	83	1.00
Rarely	13	21	0.94 (0.34, 2.56)
Sometimes	8	12	1.49 (0.45, 4.98)
Often	18	20	1.97 (0.76, 5.16)
Always	11	35	0.63 (0.23, 1.74)
Hashish			
No	15	84	1.00
Yes	81	127	2.04 (0.93, 4.47)
Heroin			
No	7	72	1.00
Yes	89	139	3.61 (1.14, 11.41)
Temgesic			
No	53	183	1.00
Yes	43	28	2.26 (1.11, 4.60)
Tramadol			
No	42	142	1.00
Yes	54	69	0.63 (0.31, 1.30)
Codeine			
No	41	150	1.00
Yes	55	61	2.54 (1.28, 5.01)
Methadone			
No	13	63	1.00
Yes	83	148	1.24 (0.51, 2.99)

Only three individuals were positive for HBsAg, so the association of potential risk factors on positive HBs Ag status could not be assessed.

## Discussion

The results of this cross-sectional study showed that the prevalence of HIV and HCV Ab positive among homeless people in Khorramabad City was considerably high and prevalence of HBsAg was lower than 1%.

Opium was the most frequently used drug and diphenoxylate had the lowest proportion of usage among the studied homeless people. Heroin, methamphetamine, and opium were the most common drugs recently used. Moreover, certain behaviors such as sharing injecting drugs, using hashish, methamphetamine and temgesic were the main risk factors associated with HIV-positive status among homeless people. The behaviors that were significantly associated with HCV positive status were the use of hashish, opium, temgesic, methamphetamine, diphenoxylate, methadone, hypnotic, and tramadol. The history of sharing injecting drugs, jail, illegal heterosexual, and homosexual activities were other main risk factors for HCV among these homeless people.

Furthermore, the prevalence of HIV (6.51%) was more than that of three other studies already conducted in Tehran including 1.7% ^12^, 3.4% ^9^, and 6.4% ^3^. According to the results of a systematic review and meta-analysis, the pooled estimation of the prevalence of HIV among male and female homeless people was 5% (95% CI: 3%, 6%) and 3% (95% CI: 2%, 5%), respectively ^13^. However, since most of the included studies in the aforementioned meta-analyses were from the developed countries, the retrieved higher HIV prevalence in this study may be due to a different epidemiologic status of HIV in Iran. In this study, about 40% of the participants had a history of drug injection and the prevalence of HIV among injecting drug users (IDUs) has been reported more than 5% in different studies in Iran ^13-15^. In our study, the prevalence of HIV among IUDs was 16.8%, on the other hand, IUDs remain at highest risk of HIV. This finding is parallel with the epidemiology of HIV in Iran where around 56% of new infection of HIV is reported among the IDUs in Iran ^16^.

In this study, using drugs such as temgesic and hypnotic was significantly associated with HIV infection. However, although the association between variables such as using methadone (OR=6.7), methamphetamine (OR=2.3), codeine (OR=2.1), and hashish (OR=1.9) and the positive status of HIV was considerable; this association was not statistically significant. Sharing injecting drugs (OR=92.3) was strongly associated with HIV infection. This result was in line with the result of another study ^9^ in Tehran (OR=8.2; 95% CI: 2.9, 23.4) among homeless people. Furthermore, the reason for not finding a significant association between using hashish, methamphetamine, methadone, and codeine with HIV infection and a wide 95% CI for the association between sharing injecting drugs and HIV infection may be due to the low number of HIV positive people and sparse data bias ^17^.

HIV infection was also associated with behavioral risk factors such as non-injecting drug usage and unsafe intercourse. The proportion of high-risk behaviors among homeless people in this study was considerable, compared to other studies in Iran ^1, 9^. On the other hand, there is an interaction between some risk factors, such as drug use, alcohol consumption, and unsafe sex and their association with HIV ^18^. In this study, 70.36% of homeless people had a history of illicit sex and 52.58% of them had not used condom during sex, so it is expected that the prevalence of HIV would increase in future. According to a prediction study in Iran, HIV infection would shift from IDUs to female sex workers (FSWs) and the trend of HIV among FSWs and men who have sex with men would increase in the future^19^.

Accordingly, the prevalence of HCV Ab positive was 31.27% in this study. This result is more than a study ^9^ conducted in Tehran (23.3%) and lower than another study conducted in the same city (42.8%) among homeless people ^3^. In a systematic review and meta-analysis, the global prevalence of HCV among male and female homeless people was 21% (95% CI: 13, 28%) and 18% (95% CI: 12, 24%), respectively ^8^. The high prevalence of HCV among homeless people in this study and other studies in Iran ^3, 9^ may be due to the high prevalence of behavioral risk factors, especially injecting drug use and needle sharing. These factors can be major contributors for the spread of HIV and viral hepatitis among this high-risk group. The prevalence of HCV in our study was higher than general population in Iran. In a population-based study in Mashhad, the prevalence of HCV was 0.13% in the general population ^20^. In Hamadan Province, the overall incidence of HCV was decreasing from 2004 to 2009, and the incidence was 5.17 per 100,000 in 2009 ^21^.

In this study, the prevalence of co-infection with HIV and HCV was 5.76%, which is more than homeless people in Tehran with 2.9% ^9^. The co-infection of HIV and HCV has some complications such as liver, immune, hematologic, renal and cardiovascular disorders for patients. In addition, the progression of HIV disease will increase in the setting of HCV co-infection ^22^.

The prevalence of the co-infection of HIV/HCV among injecting drug users in Iran was 10.95% (95% CI: 2.82, 19.08%) ^23^. Since only 38.76% of all homeless people in this study had a history of injecting drug use, the lower prevalence of co-infection was predictable in this study.

The prevalence of HBs Ag among homeless people in this study was about 1%, which is lower than that in other studies in Tehran with the prevalence of HBs Ag as 3.55 and 2.16% in 2009 and 2014, respectively ^3, 9^. However, the prevalence of HBV among injecting drug users in Iran was 3.2% ^24^. The risk of homelessness among injecting drug users is more than the risk among the general population. On the other hand, homeless people have less access to health care services, such as vaccination programs ^25, 26^. Therefore, the prevalence of HBV may increase among these high-risk groups in the near future. In Iran in 2009, the total prevalence of HBs Ag was 2.6% in the general population, and the prevalence among male and female was 3% and 2.1% respectively ^27^. The prevalence of HBs Ag in our study was lower than prevalence in male general population. A reason may be increase of the coverage of hepatitis B vaccination in the recent years in Iran. Unfortunately, in this study, we did not ask the history of HBV vaccination among the participants. However, the median age of participants was 34 yr. Therefore, the coverage of HBV vaccination among people under 34 yr may be high, because of four national HBV vaccination program in Iran from 2006 to 2010 for 15 to 18 yr age groups. In addition, the overall trend of incidence rate of HBV was decreasing over time in the west of Iran ^28^.

In addition, drug use was highly prevalent amongst homeless people in Khorramabad. Opium, methadone, and heroin were the most prevalent drugs in the past and heroin, methamphetamine, and opium were the most common drugs recently used. Addiction to some methamphetamines such as methamphetamine is associated with unsafe sex^29, 30^. Therefore, an increase in the prevalence of synthetics drugs such as methamphetamine, crack, etc., is a warning for the increase in the prevalence of HIV, viral hepatitis, and other sexually transmitted diseases among high-risk groups such as homeless people and drug users.

As for limitations of this study, some participants may actually not report their drug use status, so there is a probability of underestimation in the prevalence of certain drugs and the prevalence of other high-risk behaviors such as illicit sex and homosexuality. In addition, the participants of this study were only male homeless people, so the results were not representative to all homeless people, including female homeless people.

Hence, it is recommended that social support should be provided for these people through related governmental and non-governmental organizations. Increasing the knowledge level of homeless people on HIV and viral hepatitis is recommended. In addition, screening homeless people for HIV and viral hepatitis is also suggested in other high-risk regions of Iran in order to estimate the prevalence of these mentioned diseases and high-risk behaviors; this would lead to effective planning for interventions.

## Conclusions

The prevalence of HIV and HCV Ab positive among homeless people in this study was considerable. Moreover, the proportion of the participants who were using industrial drugs such as heroin and glass was high. With regard to the association between using drugs and HIV and HCV infections, planning participants who were using industrial drugs, such as heroin and methamphetamine, was significant for effective control and preventive interventions is crucially important amongst homeless people.

## Acknowledgements

The authors would like to thank the Iranian Center for Diseases Control and Prevention for supporting this work.

## Conflict of interest statement

The authors declare that they have no potential conflict of interest related to this study

## Funding

This study was supported by the Iranian Center for Diseases Control and Prevention.

## Highlights

- The prevalence of HIV and hepatitis C among homeless people was considerable.
- The prevalence of hepatitis B was low among homeless people.
- Abusing heroin, methamphetamine, and industrial drugs were also significant.
